# Combined nasopharyngeal and oropharyngeal sampling improves pathogen detection in community-acquired pneumonia

**DOI:** 10.3389/fmicb.2026.1774447

**Published:** 2026-04-08

**Authors:** Karin Hansen, Lisa Wasserstrom, Jonas Ahl, Yu-Ching Su, Anna C. Nilsson, Kristian Riesbeck

**Affiliations:** 1 Clinical Microbiology, Department of Translational Medicine, Faculty of Medicine, Lund University, Malmö, Sweden; 2 Infectious Diseases, Department of Translational Medicine, Faculty of Medicine, Lund University, Malmö, Sweden; 3 Department of Clinical Microbiology, Infection Control and Prevention, Skåne University Hospital, Lund, Sweden

**Keywords:** community-acquired pneumonia, *Haemophilus influenzae*, influenza virus, *Mycoplasma pneumoniae*, PCR, *Streptococcus pneumoniae*

## Abstract

**Introduction:**

Determining the etiology of community-acquired pneumonia (CAP) is essential for targeted treatment and optimal care. Reliable results require selection of the appropriate sampling site.

**Methods:**

Paired nasopharyngeal (NP) and oropharyngeal (OP) swabs were collected from 484 patients with CAP between September 2016 and September 2018. The samples were analyzed by PCRs for *Streptococcus pneumoniae*, *Haemophilus influenzae*, 6 atypical bacterial species, and 14 viral agents.

**Results:**

The most common pathogen was *S. pneumoniae* (24%), followed by *H. influenzae* (23%). Both were more frequently detected in the oropharynx, with 29% and 40% respectively, identified only in OP swabs. Respiratory viruses were found in 32% of the patients; of these, 65% were present in both NP and OP samples. Notably, 21% were detected only in NP samples, and 15% only in OP samples. *Mycoplasma pneumoniae* was identified in 6% of all patients, predominantly in OP samples (89%) compared with NP samples (70%). Cq-values were lower in NP swabs for all pathogens except *M. pneumoniae*, suggesting a higher microbial load in the nasopharynx.

**Discussion:**

This study highlights the importance of sampling site selection in the diagnosis and management of CAP. Nasopharyngeal sampling was more effective for detecting respiratory viruses, whereas OP samples yielded more cases of *S. pneumoniae*, *H. influenzae* and *M. pneumoniae*. To maximize the diagnostic yield, a combined NP/OP-sampling strategy can thus be recommended.

## Introduction

Community-acquired pneumonia (CAP) is one of the most common infectious diseases and globally a major cause for morbidity and mortality (GBD 2016 Lower Respiratory Infections Collaborators, 2018). The annual incidence of hospitalization due to CAP in European and American studies ranges between 206 and 649 per 100,000 (Bjarnason et al., 2018; Jain et al., 2015; Ramirez et al., 2017; Søgaard et al., 2014). The primary causative pathogen of CAP has historically been *Streptococcus pneumoniae*, but studies indicate an increasing incidence of *Haemophilus influenzae* (Fally et al., 2021; Gadsby and Musher, 2022; Yamba Yamba et al., 2024). *Mycoplasma pneumoniae* occurs in epidemics and, although more frequent in children and ambulatory patients, can also cause hospitalization in adults (Khoury et al., 2016). Viruses are a common finding in CAP, both in combination with bacteria and as the sole cause of CAP (Jain et al., 2015). Determining the infectious etiology is important for the targeted use of antimicrobial agents, as well as for implementing infection prevention and control measures.

The reliability of a diagnostic test, however, depends on the quality of the sample. Sampling from the lower respiratory tract is preferred in CAP-patients (Metlay et al., 2019), but an upper respiratory tract (URT) sample is more easily obtained in the emergency room. Studies on URT sampling for viruses in adults have primarily focused on influenza virus, demonstrating similar or higher yields from nasopharyngeal (NP) swabs compared to oropharyngeal (OP) swabs (Hernes et al., 2013; Spencer et al., 2013). With the availability of rapid syndromic testing for a spectrum of respiratory pathogens including both viral and bacterial agents (Poole and Clark, 2020), studies comparing sampling sites have typically contrasted sputum with either OP or NP samples, or with dual sampling (Bjarnason et al., 2017; Nyawanda et al., 2019; Serigstad et al., 2023). This study aimed to compare NP and OP swabs for detection of various respiratory pathogens, including *S. pneumoniae*, *H. influenzae*, atypical bacteria including *M. pneumoniae*, and common respiratory viruses to determine the preferred method for URT respiratory sampling.

## Materials and Methods

### Patients and sampling

This study was conducted at Skåne University Hospital (Malmö, Sweden) between September 2016 and September 2018. Patients hospitalized with predefined symptoms of respiratory infection and radiological signs of pneumonia were screened for inclusion. Patients who had been admitted to the hospital within the last 30 days were excluded. Patient samples were from the ECAPS (Etiology of community acquired pneumonia in Sweden) study, and the inclusion and exclusion criteria are described in detail elsewhere (Hansen et al., 2023a). Briefly, after obtaining informed consent, paired flocked swabs (Sigma VCM, MWE, Corsham, UK product code: MW911PF2ML and MW910PF) were collected by one of the 5 trained research nurses from both the nasopharynx and oropharynx. Sampling was performed within 48 h of presenting to the emergency room and in accordance with local guidelines, the NP sample was retrieved by inserting the swab through the nostril to the nasopharynx rotating the swab 360 degrees and waiting 5 s before extracting the swab (Region SKANE, 2024b). The OP samples was collected by inserting the swab, lightly scraping and pressing the oropharynx and tonsils, then stroking the oropharynx three times, retracting the swab avoiding contact with the tongue or mucosa (Region SKANE, 2024a). The swabs were transferred to a universal transport medium, the swabs were rotated, pressed against the edge to extract fluid and then removed from the medium that was frozen at −80 °C.

### Nucleic acid testing (NAT) for viral agents

All samples were analyzed at Clinical Microbiology, Laboratory Medicine Skåne (Lund, Sweden), a laboratory that is accredited according to the ISO 15189 standard. Extraction of DNA/RNA was performed from a 200 μl sample without pretreatment, using the MagNA Pure 96 DNA and Viral NA Small volume kit (Roche Diagnostics, Basel, Switzerland) with addition of 20 μl poly(A) (Roche Diagnostics) per sample. The final elution volume was 100 μl. Multiplex real-time PCRs were performed to detect 14 respiratory viruses (influenza A H1N1, influenza A H3N2, influenza B, enterovirus, rhinovirus, parechovirus, adenovirus, human metapneumovirus (hMPV), and coronaviruses (OC43, NL63, 229E), as well as parainfluenza virus (PIV) 1–3 and respiratory syncytial virus (RSV) A/B. The PCRs could not always discriminate between rhinovirus and enterovirus, and these are therefore reported as one group.

### NAT for bacteria

Real-time PCR was used to identify the atypical bacterial pathogens *Bordetella pertussis*, *B. parapertussis*, *Chlamydia pneumoniae*, and *Mycoplasma pneumoniae*, and, finally, *S. pneumoniae* and *H. influenzae*. The target gene for *S. pneumoniae* was *lytA*. For *H. influenzae* all OP and NP swabs were tested with a PCR targeting *glpQ* [synonym *hpd*, encoding surface lipoprotein glycerophosphodiester phosphodiesterase (Protein D) (Forsgren et al., 2008)]. Due to many positive results for *H. influenzae* in the OP site using forward (5′-CTGGWGCAATGGCAGAAGTG-3′; 500 nM) and reverse (5′-TCTTTACGCACGGTGTAAGGATG-3′; 500 nM) primers and probe (5′-[6-FAM]-AATATGCCGATGGTGTTGGYCCAGGTT-[TAMRA]-3′; 100 nM) from Smith-Vaughan et al. (2006), all OP samples were also tested with an additional PCR with forward (5′-GGTTAAATATGCCGATGGTGTTG-3′; 100 nM) and reverse primers (5′- TGCATCTTTACGCACGGTGTA-3′; 300 nM) and probe (5′-[FAM]-TTGTGTACACTCCGT/ ZEN/TGGTAAAAGAACTTGCAC-[BHQ1]-3′; 100 nM) targeting another part of the *glpQ/hpd* gene (*hpd*#3) being more specific for *H. influenzae* (de Gier et al., 2016). This enabled further discrimination of *H. haemolyticus* from *H. influenzae*. In addition, all NP samples with any reactivity with primers and probe from Smith-Vaughan et al. (2006) (*glpQ/hpd*) were tested with *hpd*#3 to exclude false positive signals revealing *H. haemolyticus*. For atypical bacteria and *S. pneumoniae*, a cut-off value of Cq < 39 was applied, and for *H. influenzae* Cq < 38 was used. The detailed PCR protocols for all pathogens except for *H. influenzae* have been previously described (Hansen et al., 2023b).

### Statistics

Data analysis was performed using IBM SPSS statistics version 29.0.0.0. Differences in OP- and NP sampling sites were analyzed with McNemar’s test and Kappa statistics. Respiratory viruses were compared as a group or individual viruses. Differences in Cq-values were estimated using Wilcoxon signed ranked test, *p*-values < 0.05 were considered significant. Sensitivity was calculated using a positive test in either sample as true positive.

## Results

### Patient characteristics

From a cohort of 518 patients hospitalized with CAP, paired NP and OP swabs were collected from 484 patients. The median age was 73 years, and 44% was female. The median symptom duration at the time of sampling was 4 days and 20% of the patients had received antibiotics prior to presenting to the Emergency Department. Patient characteristics are outlined in Table 1.

**TABLE 1:** Clinical characteristics of CAP patients.

Variable	*n* = 484
Age, median [IQR*^a^*]	73 [61, 83]
Female sex, *n* (%)	211 (44)
Co-morbidities, *n* (%)	
COPD	137 (28)
Congestive heart failure	88 (18)
Coronary heart disease	127 (26)
Diabetes mellitus	80 (17)
Immunodeficiency*^b^*	7 (1)
Malignancy	123 (25)
Asthma	44 (9)
ICU admission, *n* (%)	13 (3)
CFR*^c^* 30 days, *n* (%)	18 (4)
Length of stay, median [IQR*^a^*]	5.0 [3.0, 8.0]
Symptom duration*^d^*, median [IQR*^a^*]	4.0 [1.3, 7.0]
Immunization status	
Seasonal influenza, *n* (%)	163 (34)
*Streptococcus pneumoniae^e^*, *n* (%)	53 (11)

*^a^*Inter-quartile range.

*^b^*Including primary immunodeficiency disorders, HIV/AIDS and organ transplanted patients.

*^c^*Case-fatality rate.

*^d^*Symptom duration before sampling.

*^e^*PCV13 (Prevenar13^®^) or PPV23 (Pneumovax^®^).

### Site-specific detection of key respiratory bacteria

*Streptococcus pneumoniae* was the most frequently detected individual pathogen, identified in 118 patients (24%), with no significant difference between sampling sites (*p* = 0.141) (Table 2). There were 34 positives of *S. pneumoniae* (29%) in OP swabs only, compared to 22 positives (19%) in NP-samples only. The second most frequently detected agent was *H. influenzae*, found in 109 patients (23%), out of which 40% were only detected in OP swabs compared to 6% only in NP-samples (*p* < 0.001). *Mycoplasma pneumoniae* was identified in 27 patients, of whom 8 cases (30%) were positive only in OP samples compared to 3 cases (11%) only in NP.

**TABLE 2:** Distribution of detected pathogens by swab type.

Pathogens detected in	NP only	OP only	NP and OP	Total	NP sensitivity^a^ (CI 95%)	OP sensitivity^a^ (CI 95%)	*P*-value	Kappa statistics^b^ (CI 95%)
Bacteria, *n* (%)^c^								
*S. pneumoniae*	22 (19)	34 (29)	62 (53)	118	0.71 (0.62–0.79)	0.81 (0.73–0.87)	0.141	0.62 (0.53–0.71)
*H. influenzae*	7 (6)	44 (40)	58 (53)	109	0.59 (0.49–0.68)	0.94 (0.87–0.97)	**<0.001**	0.64 (0.54–0.73)
*M. pneumoniae*	3 (11)	8 (30)	16 (59)	27	0.70 (0.52–0.85)	0.89 (0.73–0.97)	0.227	0.73 (0.58–0.88)
Viruses, *n* (%)								
Influenza A^d^	4 (21)	6 (32)	9 (47)	19	0.68 (0.43–0.87)	0.79 (0.54–0.94)	0.754	0.63 (0.42–0.84)
Influenza B	2 (9)	3 (13)	18 (78)	23	0.87 (0.66–0.97)	0.91 (0.73–0.99)	1.000	0.87 (0.76–0.98)
hMPV	8 (30)	1 (4)	18 (67)	27	0.96 (0.81–1.0)	0.70 (0.50–0.86)	**0.039**	0.79 (0.66–0.92)
Rhino/Enterovirus	10 (18)	7 (13)	38 (69)	55	0.87 (0.76–0.95)	0.82 (0.69–0.91)	0.629	0.80 (0.71–0.89)
PIV 1–3	1 (10)	3 (30)	6 (60)	10	0.70 (0.35–0.93)	0.90 (0.56–1.0)	0.625	0.75 (0.51–0.99)
Coronavirus OC43/NK63/229E	5 (56)	0 (0)	4 (44)	9	1.0 (0.66–1.0)	0.44 (0.14–0.79)	0.063	0.61 (0.30–0.92)
RSV	2 (17)	1 (8)	9 (75)	12	0.86 (0.62–0.97)	0.79 (0.53–0.94)	1.000	0.85 (0.70–1.02)
Adenovirus	1 (50)	1 (50)	0	2	n/a	n/a	n/a	n/a
Total	33 (21)	22 (15)	102 (65)	157	0.86 (0.90–0.91)	0.79 (0.72–0.85)	0.229	0.71 (0.64–0.78)

*^a^*Sensitivity calculated using a positive sample in either sampling location as true positive.

*^b^*Kappa level of agreement: >0.8 almost perfect, >0.6 substantial, >0.4 moderate, >0.2 fair, >0 slight, <0 no agreement.

*^c^*One case of *C. pneumoniae* and one *B. parapertussis*, both positive only in NP-sample. No positive case of *B. pertussis*.

*^d^*Four patients with H1N1 and 15 patients with H3N2. *P*-values < 0.05 are indicated in bold.

### Respiratory virus detection across sampling sites

A total of 157 respiratory viruses were detected in either sampling site in 153 individual patients (32%). Three patients tested positive for more than one virus. Of all viral findings, 65% were concordant between nasopharyngeal and oropharyngeal samples. In 22% of cases, viruses were found only in NP samples and in 15% only in OP samples. The most detected viruses, in descending order, were rhino/enterovirus, influenza A/B and hMPV (Table 2). For influenza viruses, 6 cases (14%) were detected only in NP samples and 9 (21%) only in OP samples.

### Pathogen load and symptom duration in concordant and discordant samples

**FIGURE 1:**
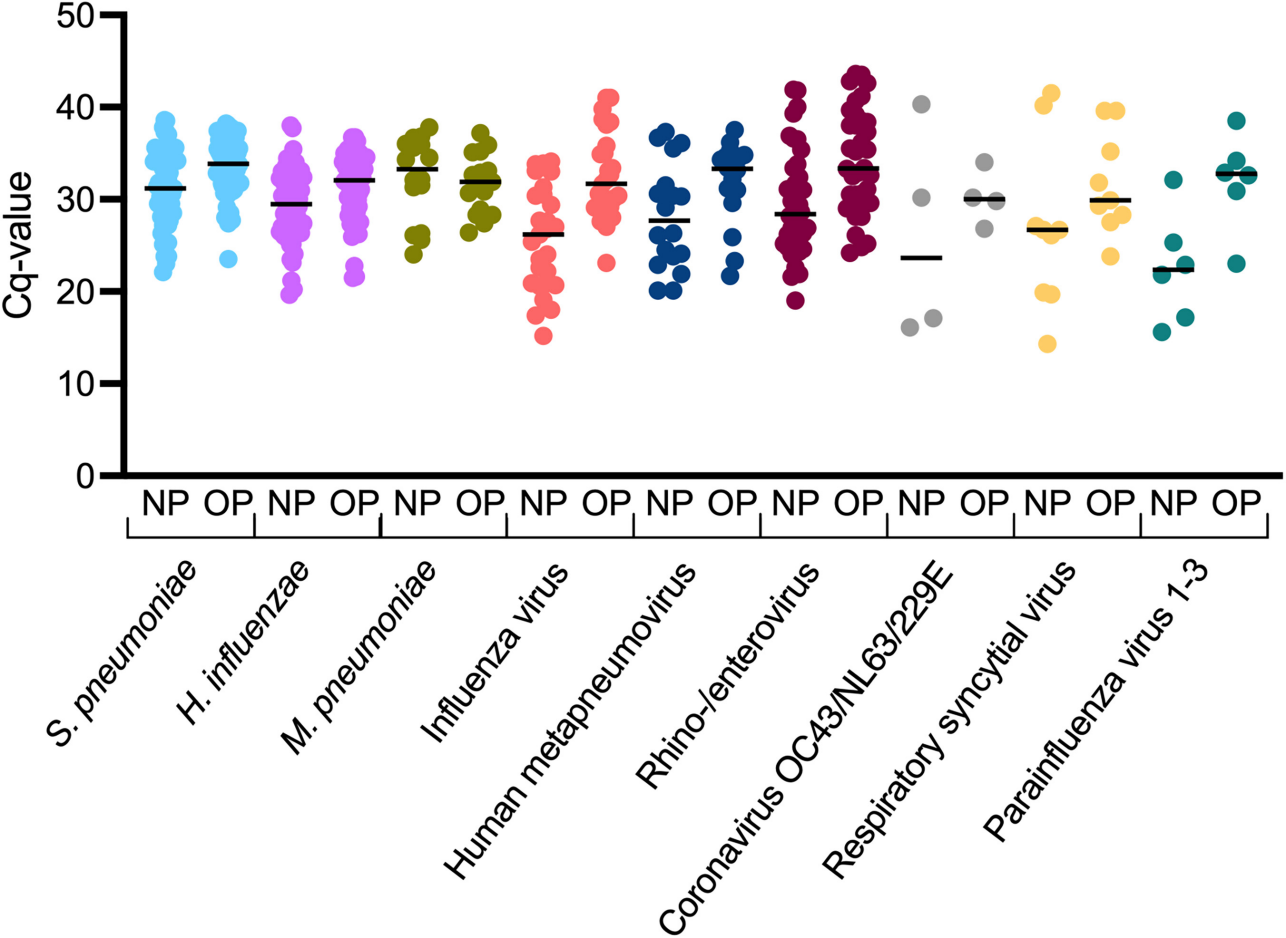
Comparison of Cq-values for different pathogens in nasopharyngeal (NP) and oropharyngeal (OP) samples. Samples which were positive in both NP and OP swabs are shown (concordant samples). Each dot represents an individual Cq-value, and lines represent the median Cq-values.

**FIGURE 2:**
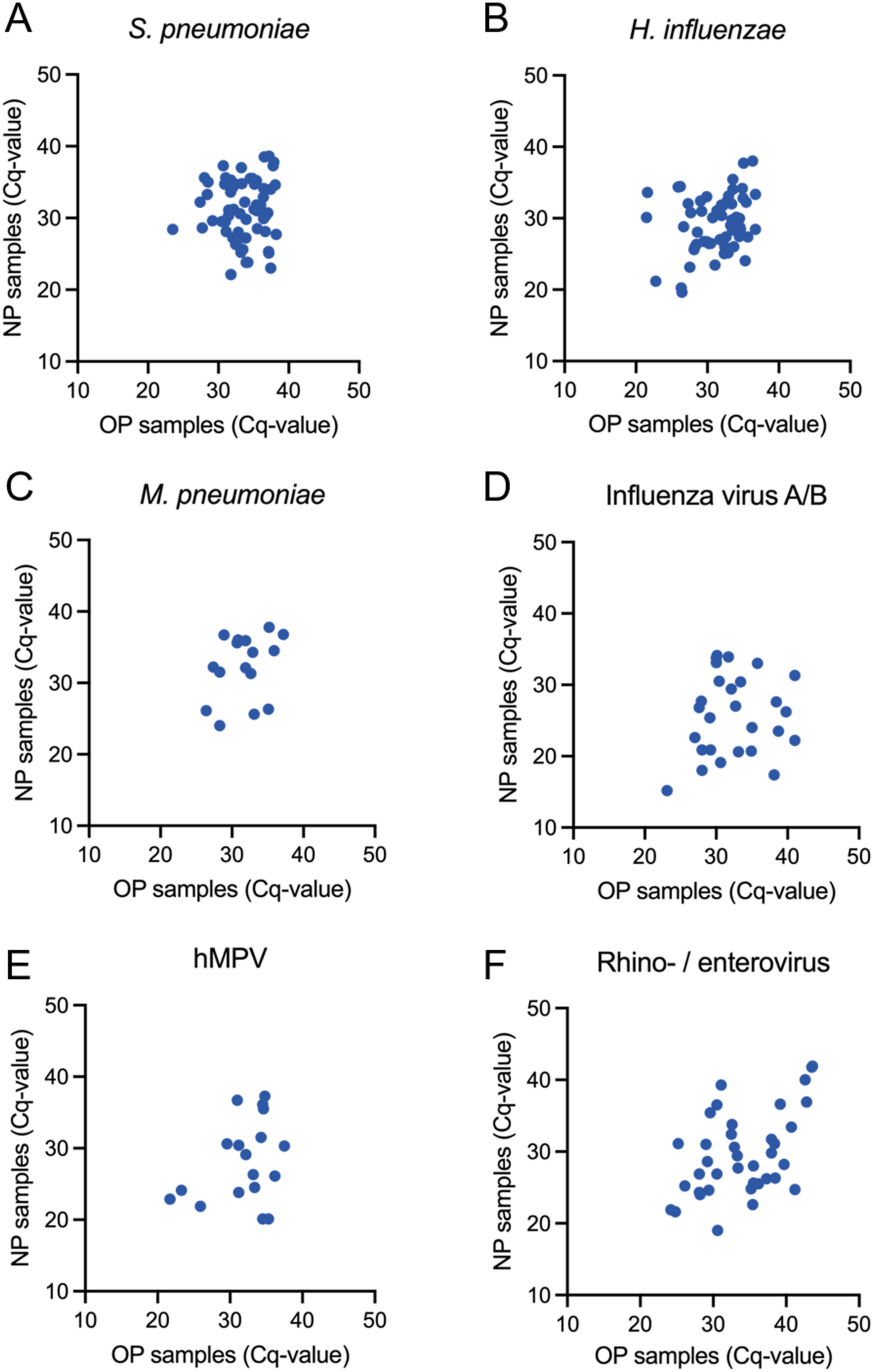
Detailed analyses of Cq-values in nasopharyngeal (NP) and oropharyngeal (OP) swabs for patients positive in both samples. Each dot represents the same patient’s Cq-values in respective swab. **(A)** *Streptococcus pneumoniae*, **(B)** *Haemophilus influenzae*, **(C)** *Mycoplasma pneumoniae*, **(D)** influenza virus A/B, **(E)** human metapneumovirus (hMPV), and **(F)** Rhino- and enterovirus are indicated. Respiratory syncytial virus, Corona virus and Parainfluenza virus 1–3 are not shown due to few positive samples.

Mean Cq-values for samples positive in both swabs were lower in the NP-swabs for *S. pneumoniae*, *H. influenzae* and all viruses, suggesting a higher pathogen load in the nasopharynx compared to the oropharynx (Figure 1 and Table 3). For *M. pneumoniae*, the Cq-values were lower in OP samples, although the difference was not statistically significant (Figure 2 and Table 3). Discordant samples showed higher mean Cq-values for all pathogens except *M. pneumoniae*, compared with concordant samples, indicating a lower pathogen load in patients with discordant results. Interestingly, for *S. pneumoniae*, *H. influenzae* and *M. pneumoniae*, patients with only one positive sample in either OP or NP swab had a shorter symptom duration before sampling compared to those with two positive samples: a median of 3 versus 5 days for *S. pneumoniae*, 3 versus 6 days for *H. influenzae* and 6 versus 7 days for *M. pneumoniae*. In contrast, among viral infections, patients with only one positive sample had a longer median symptom duration (6 days) compared to those with two positive samples (4 days).

**TABLE 3:** Comparison of Cq-values in different sample sites.

Mean Cq-values	NP^a^	OP^a^	*P*-value	Only NP positive^b^	Only OP positive^b^
*S. pneumoniae*	31.2	33.6	**0.002**	34.0	35.4
*H. influenzae*	28.9	30.8	**0.001**	32.4	33.7
*M. pneumoniae*	32.2	31.3	0.485	31.6	31.3
Influenza	25.8	32.5	**<0.001**	31.4	37.2
hMPV	28.1	32.0	**0.037**	33.1	34.4^d^
Rhino/enterovirus	29.6	34.0	**<0.001**	33.3	35.1
Coronavirus^c^	25.9	30.2	0.465	26.9	N/A
RSV	27.0	31.7	**0.028**	31.5	37.7
PIV 1–3	22.5	32.0	**0.028**	26.3^d^	32.5

*^a^*Mean value calculated from samples positive in both specimens.

*^b^*Mean values calculated from the discordant specimens.

*^c^*OC43/NK63/229E.

*^d^*Only one sample. *P*-values < 0.05 are indicated in bold.

## Discussion

This study, focusing on the optimal URT-sampling site for comprehensive PCR-testing of respiratory pathogens in CAP, showed relatively good agreement between the NP- and OP-specimens. However, sampling from single anatomical sites resulted in undiagnosed cases. Different pathogens were more likely to have distinct optimal sampling sites, which is important to consider when interpreting a negative result and when to choose which sample type depending on which pathogen is tested for. This further supports the use of combined NP and OP swabs.

*Streptococcus pneumoniae* was the most frequently detected pathogen. We found a higher detection rate in the oropharynx, albeit not statistically significant, which contrasts with a study that compared different URT samples. In that study, 13 patients with a lower respiratory tract infection (LRTI) were culture positive for *S. pneumoniae* in OP samples versus 29 in NP-samples (Lieberman et al., 2006). Another study investigating pneumococcal carriage in adults in Kenya found more pneumococci in nasopharyngeal cultures than samples from oropharynx (Farrar et al., 2020). However, there are two important considerations regarding the detection of pneumococci in the URT, first, the specificity of the target gene, in this case *lytA*, and second, the challenge of distinguishing colonization from actual infection. Although the target gene *lytA* has a high specificity for *S. pneumoniae*, it is also found in other streptococci from the oral microbiome (Abdeldaim et al., 2010). Several studies have compared sputum samples with various URT samples using PCR targeting *lytA*. One study comparing sputum, NP swab and nasopharyngeal aspirates reported a sensitivity of 61% and specificity of 89% for NP samples analysed by PCR, compared with 86% sensitivity and 66% specificity for sputum culture when using a positive pneumococcal urinary antigen test or blood culture as a marker of definite pneumococcal etiology (Strålin et al., 2006). Nyawanda et al. (2019) compared pooled OP and NP samples with sputum in patients with acute lower respiratory infection and found a slightly higher rate of *S. pneumoniae* in the URT-samples; 71 vs. 61 cases. Thus, an URT- sample may be a suitable option for establishing pneumococcal etiology in patient groups with an expected low colonization rate, although the specificity of OP-swabs is possibly lower than that of NP-swabs.

In our study using real-time PCR, *H. influenzae* was mostly detected in OP samples. Only a limited number of studies have addressed *H. influenzae* in relation to sampling site; for example, a study of 300 LRTI patients using culturing, growth of *H. influenzae* was reported in 13 OP samples compared with 8 NP samples (Lieberman et al., 2006). Another study on *H. influenzae* carriage in healthy mothers reported that 81% of culture positive samples were detected exclusively in OP samples. This may not, however, be applicable on symptomatic patients. Furthermore, different gene targets for *H. influenzae* PCR present different challenges with respect to specificity and potential cross-reactivity with other non-pathogenic *Haemophilus* spp. such as *H. haemolyticus* (Pickering et al., 2014). *Haemophilus parainfluenzae* and *H. aegyptius* can also present challenges in cross-reactivity, especially as *H. parainfluenzae*, although it only causes disease in rare cases, (Oill et al., 1979) has been found in abundance in the oral microbiome (Caselli et al., 2020). The relatively rare *H. aegyptius* is known to cause bilateral conjunctivitis (Steinhoff and Cherian, 2006) and has been linked to outbreaks of purpuric fever in Brazil (Brazilian Purpuric Fever Study Group, 1987). This study used an adapted PCR to reduce such cross-reactivity, but it could still occur to some extent. The possibility of cross-reactivity for both *S. pneumoniae* and *H. influenzae* with other closely related species in the oral microbiome should be taken into consideration when using multiplex assays, especially if the gene targets are not provided, and in choosing which sampling site to use. More research is thus needed in syndromic testing on URT-samples for *S. pneumoniae* and *H. influenzae*.

Among the 27 cases harboring *M. pneumoniae*, almost one third (8/27) were detected only in OP samples, compared with 3/27 only in NP samples, indicating a lower sensitivity of the nasopharyngeal site in this cohort. There was no difference in Cq values between concordant positive and discrepant samples, and the discrepant samples did not show higher Cq-values, this suggests that the missed cases did not have a lower bacterial load. Only one larger study has compared sampling sites with OP being superior to NP-testing. In that study, including only pediatric patients, 43 cases out of 182 positive samples were positive only in the OP-samples compared to 6 cases in the NP-swabs (Kitagawa et al., 2025). Other studies have had more varying results but with small case numbers (between 7 and 18) (Dorigo-Zetsma et al., 2001; Gnarpe et al., 1997; Kakuya et al., 2017; Michelow et al., 2004).

For respiratory viruses, 35% of the results were discordant, with a higher number of positive samples in NP swabs. Adding the OP swab to the NP swab increased the overall viral detection rate by 16%. Significantly lower Cq values were observed in NP-samples for most viruses, suggesting a higher viral load in the nasopharynx, as previously demonstrated in the same setting (Ek et al., 2019). Other studies have also demonstrated that inconsistent results between NP and OP-sampling are associated with higher Cq-values, indicating lower viral RNA levels in specimens only detected in one sampling site compared to concordant pairs (Spencer et al., 2013). Additionally, the duration of symptoms before sampling was longer in patients with only one positive swab, suggesting a decrease in viral load over time.

Influenza virus is particularly important to detect promptly so that antiviral treatment can be initiated and to mitigate the high risk of hospital transmission. In our study, 9 out of 42 cases were missed when using NP sampling alone, and 6 out of 42 were missed when using OP sampling alone. A study predominantly involving children, with 321 positive influenza cases, observed difference in influenza sub-type detection: nasopharyngeal sampling detected more influenza B cases, whereas OP sampling were superior for detecting influenza A, primarily H1N1 (Kim et al., 2011). In contrast, we found that OP sampling detected more cases of both influenza A and B, with only few cases of H1N1. Other authors found no significant difference between OP and NP sampling (Ek et al., 2019; Spencer et al., 2013). Two additional studies reported higher sensitivity of NP sampling for detecting influenza A. The study by Lieberman et al. (2009) involved hospitalized patients with lower respiratory infections and was conducted prior to the H1N1 pandemic. These authors also used cotton-tip swabs which could have affected the total yield as flocked swabs have shown a higher sensitivity (Hernes et al., 2011). In contrast, Hernes et al. (2013) focused on patients already known to be influenza-positive, and the initial sampling method was not specified. For viruses, the agreement between OP and NP samples was weaker in patients with longer duration of symptoms, whereas for bacteria it was stronger, which may indicate a decreasing viral load and an increasing bacterial load with time.

The strengths of our study are the prospective design, the limited number of personnel involved in sample collecting, and the use of flocked swabs for both samples. However, there are a few limitations. Some viruses were detected in low numbers, and the sensitivity analysis is suboptimal due to the absence of a gold standard or simultaneous lower respiratory tract specimens. Furthermore, the samples were collected after antibiotic administration which, although PCR also detects dead bacteria, could affect the bacterial load. Twenty percent of the patients had been prescribed antibiotics prior to their hospital visit, which has previously been shown to be associated with a reduction in PCR detection (Saukkoriipi et al., 2015). In samples where *S. pneumoniae* and *H. influenzae* were detected the Cq-values were consistently lower in NP-swabs although these microbes were more frequently detected in the OP-samples. This pattern may indicate cross-detection related to the composition of the oropharyngeal microbiome. Finally, as this study was restricted to patients with CAP and the median age in the cohort was 73 years, the findings may not be directly generalizable to younger populations or to individuals with upper respiratory tract infections.

In conclusion, when sampling from the upper respiratory tract in patients with CAP, there is a risk of false-negative results, and the optimal sampling site may vary depending on the pathogen. Combined OP/NP swabbing is preferable in CAP patients, particularly when using multiplex panels. Further studies are needed to evaluate the specificity of *S. pneumoniae* and *H. influenzae* PCRs.

## Data Availability

The raw data supporting the conclusions of this article will be made available by the authors, without undue reservation.
